# Supplementary material for: Novel anti-virulence compounds disrupt exotoxin expression in MRSA
Source: Microbiol Spectr. 2024 Oct 21;12(12):e01464-24. doi: 10.1128/spectrum.01464-24 (PMC11619317; doi:10.1128/spectrum.01464-24)
Supplement: Supplemental figures and tables — Fig. S1 to S8; Tables S1 to S3 and S5. [file spectrum.01464-24-s0001.pdf]

Supporting Information

Novel Anti-virulence Compounds Disrupt Exotoxin Expression in MRSA

Balogh, Halie[a], Anthony, Amaiya[b],Stempel, Robin[a], Vossen, Lauren[b], Federico, Victoria A.[b], Valenzano, Gabriel Z.[a], Blackledge, Meghan S.[a]*, and Miller, Heather B.[a]*

Author Affiliations:
[a]Department of Chemistry, High Point University, High Point, North Carolina 27268, United States
[b]Department of Biology, High Point University, High Point, North Carolina 27268, United States
*Corresponding Authors: Meghan S. Blackledge mblackle@highpoint.edu, Heather B. Miller hmiller@highpoint.edu

Page

Supporting Tables

S1 – Additional quality control details of USA100 RNA samples 2
S2 – RNA-seq read quality control 3
S3 – Mapped reads summary 4
S4 – All differential gene expression results for USA100 (.xlsx)
S5 – RT-qPCR primer information 5

Supporting Figures

S1 – Alpha hemolysin precursor alignment 6
S2 – Beta hemolysin alignment 6
S3 – Gamma hemolysin component A alignment 7
S4 - Gamma hemolysin component B alignment 7
S5 - Gamma hemolysin component C alignment 7
S6 – Delta hemolysin precursor alignment 8
S7 - RT-qPCR validation of RNA-seq determined gene expression changes 8
S8 – Full western blot images 9

References 10

Table S1: Additional quality control details of USA100 RNA samples. Un represents untreated sample, ox represents oxacillin treated sample, 8 represents compound 8 treated sample, lor represents loratadine treated sample, 8ox represents a cotreated sample, and lorox represents a cotreated sample. Each biological replicate is labelled A, B, or C. RIN= RNA integrity number.

Sample #	Sample ID	Customer Sample ID	Sample Type	Sample Volume (ul)	Concentration (ng/ul)	Total Quantity (ng)	RIN
1	1735R-2069-01	USA100unA	RNA	53	170.9	9055.9	7.6
2	1735R-2069-02	USA100oxA	RNA	53	44.3	2346.0	7.6
3	1735R-2069-03	USA1008A	RNA	52	57.1	2968.5	7.1
4	1735R-2069-04	USA100lorA	RNA	52	123.4	6418.8	7.3
5	1735R-2069-05	USA1008oxA	RNA	52	150.6	7830.2	7.2
6	1735R-2069-06	USA100loroxA	RNA	65	8.8	573.7	NA
7	1735R-2069-07	USA100unB	RNA	52	370.6	19271.1	7.9
8	1735R-2069-08	USA100oxB	RNA	52	28.0	1456.4	7.1
9	1735R-2069-09	USA1008B	RNA	52	85.6	4452.5	7.0
10	1735R-2069-10	USA100lorB	RNA	52	97.6	5076.5	6.8
11	1735R-2069-11	USA1008oxB	RNA	52	39.2	2040.7	7.5
12	1735R-2069-12	USA100loroxB	RNA	52	55.2	2869.6	6.7
13	1735R-2069-13	USA100unC	RNA	51	267.5	13640.8	8.1
14	1735R-2069-14	USA100oxC	RNA	48	6.7	322.0	NA
15	1735R-2069-15	USA1008C	RNA	51	41.8	2134.0	7.2
16	1735R-2069-16	USA100lorC	RNA	51	103.5	5280.3	7.3
17	1735R-2069-17	USA1008oxC	RNA	51	146.1	7448.8	7.3
18	1735R-2069-18	USA100loroxB	RNA	48	5.5	264.8	NA

Table S2: RNA-seq reads quality control details. Un represents untreated sample, ox represents oxacillin treated sample, Cmpd8 represents compound 8 treated sample, Lor represents loratadine treated sample, Cmpd8_Ox represents cotreated sample, and Lor_Ox represents cotreated sample. Each biological replicate is labelled A, B, or C. Q20 and Q30 were calculated as the base number of Phred value > 20 or 30, respectively, divided by the total base value x 100%.

Sample name	Raw reads	Clean reads	Raw bases	Clean bases	Error rate	Q20	Q30	GC content
Un_A	16563404	16199626	2.49G	2.43G	0.03	97.55	93.01	34.95
Ox_A	16407200	15973518	2.47G	2.4G	0.03	97.61	93.16	35.00
Cmpd8_A	17782084	17355948	2.67G	2.61G	0.03	97.57	93.06	35.38
Lor_A	17763720	17237008	2.67G	2.59G	0.03	97.63	93.18	34.99
Cmpd8_Ox_A	18616198	18165322	2.8G	2.73G	0.03	97.57	93.05	35.30
Lor_Ox_A	17264464	16941150	2.59G	2.55G	0.03	97.59	93.1	34.94
Un_B	18606480	18109918	2.8G	2.72G	0.03	97.49	92.87	35.13
Ox_B	16118996	15664202	2.42G	2.35G	0.03	97.59	93.1	34.79
Cmpd8_B	17760996	17308642	2.67G	2.6G	0.03	97.68	93.3	35.48
Lor_B	16010694	15431796	2.41G	2.32G	0.03	97.71	93.37	35.01
Cmpd8_Ox_B	14668862	14310466	2.21G	2.15G	0.03	97.69	93.32	35.25
Lor_Ox_B	13825412	13380550	2.08G	2.01G	0.03	97.8	93.65	34.85
Un_C	14488574	14014188	2.18G	2.11G	0.03	97.64	93.19	34.75
Ox_C	14465062	13989522	2.17G	2.1G	0.03	97.75	93.53	35.28
Cmpd8_C	14354250	13838842	2.16G	2.08G	0.03	97.76	93.5	34.97
Lor_C	12894550	12528502	1.94G	1.88G	0.03	97.74	93.45	34.87
Cmpd8_Ox_C	15374400	14948966	2.31G	2.25G	0.03	97.61	93.2	35.41
Lor_Ox_C	13252778	12708422	1.99G	1.91G	0.03	97.56	93.26	36.32

Supporting Table S3: Mapped reads summary. Un represents untreated sample, ox represents oxacillin treated sample, Cmpd8 represents compound 8 treated sample, Lor represents loratadine treated sample, Cmpd8_Ox represents cotreated sample, and Lor_Ox represents cotreated sample. Each biological replicate is labelled A, B, or C.

**Sample name**	**Cmpd8_A**	**Cmpd8_B**	**Cmpd8_C**	**Cmpd8_Ox_A**	**Cmpd8_Ox_B**	**Cmpd8_Ox_C**	**Lor_A**	**Lor_B**	**Lor_C**
Total reads	17355948	17308642	13838842	18165322	14310466	14948966	17237008	15431796	12528502
Total mapped	17124337 (98.67%)	16745919 (96.75%)	13632347 (98.51%)	17917673 (98.64%)	13821016 (96.58%)	14726483 (98.51%)	17004296 (98.65%)	14877913 (96.41%)	12354208 (98.61%)
Multiple mapped	653694 (3.77%)	916949 (5.3%)	501832 (3.63%)	930892 (5.12%)	723524 (5.06%)	837940 (5.61%)	572647 (3.32%)	683508 (4.43%)	426828 (3.41%)
Uniquely mapped	16470643 (94.9%)	15828970 (91.45%)	13130515 (94.88%)	16986781 (93.51%)	13097492 (91.52%)	13888543 (92.91%)	16431649 (95.33%)	14194405 (91.98%)	11927380 (95.2%)
Read-1	8240255 (47.48%)	7922309 (45.77%)	6567562 (47.46%)	8498666 (46.79%)	6554086 (45.8%)	6947681 (46.48%)	8220857 (47.69%)	7102905 (46.03%)	5966217 (47.62%)
Read-2	8230388 (47.42%)	7906661 (45.68%)	6562953 (47.42%)	8488115 (46.73%)	6543406 (45.72%)	6940862 (46.43%)	8210792 (47.63%)	7091500 (45.95%)	5961163 (47.58%)
Reads map to '+'	8236092 (47.45%)	7917616 (45.74%)	6566198 (47.45%)	8494025 (46.76%)	6551387 (45.78%)	6944313 (46.45%)	8216744 (47.67%)	7099985 (46.01%)	5964315 (47.61%)
Reads map to '-'	8234551 (47.45%)	7911354 (45.71%)	6564317 (47.43%)	8492756 (46.75%)	6546105 (45.74%)	6944230 (46.45%)	8214905 (47.66%)	7094420 (45.97%)	5963065 (47.6%)
Reads mapped in proper pairs	15178748 (87.46%)	14427716 (83.36%)	12207224 (88.21%)	15629750 (86.04%)	12020118 (84%)	12626678 (84.47%)	15282116 (88.66%)	13020262 (84.37%)	11087146 (88.5%)
Proper-paired reads map to different chrom	0 (0%)	0 (0%)	0 (0%)	0 (0%)	0 (0%)	0 (0%)	0 (0%)	0 (0%)	0 (0%)

**Sample name**	**Lor_Ox_A**	**Lor_Ox_B**	**Lor_Ox_C**	**Ox_A**	**Ox_B**	**Ox_C**	**Un_A**	**Un_B**	**Un_C**
Total reads	16941150	13380550	12708422	15973518	15664202	13989522	16199626	18109918	14014188
Total mapped	16619486 (98.1%)	12903635 (96.44%)	12391696 (97.51%)	15701622 (98.3%)	15063006 (96.16%)	13692023 (97.87%)	15995883 (98.74%)	17455039 (96.38%)	13830339 (98.69%)
Multiple mapped	888045 (5.24%)	642245 (4.8%)	1468567 (11.56%)	634985 (3.98%)	718424 (4.59%)	929260 (6.64%)	494791 (3.05%)	1025989 (5.67%)	412861 (2.95%)
Uniquely mapped	15731441 (92.86%)	12261390 (91.64%)	10923129 (85.95%)	15066637 (94.32%)	14344582 (91.58%)	12762763 (91.23%)	15501092 (95.69%)	16429050 (90.72%)	13417478 (95.74%)
Read-1	7869562 (46.45%)	6135011 (45.85%)	5464181 (43%)	7536052 (47.18%)	7176663 (45.82%)	6382457 (45.62%)	7754299 (47.87%)	8222475 (45.4%)	6711588 (47.89%)
Read-2	7861879 (46.41%)	6126379 (45.79%)	5458948 (42.96%)	7530585 (47.14%)	7167919 (45.76%)	6380306 (45.61%)	7746793 (47.82%)	8206575 (45.32%)	6705890 (47.85%)
Reads map to '+'	7867176 (46.44%)	6133063 (45.84%)	5461995 (42.98%)	7535189 (47.17%)	7173192 (45.79%)	6382866 (45.63%)	7751824 (47.85%)	8216554 (45.37%)	6709875 (47.88%)
Reads map to '-'	7864265 (46.42%)	6128327 (45.8%)	5461134 (42.97%)	7531448 (47.15%)	7171390 (45.78%)	6379897 (45.6%)	7749268 (47.84%)	8212496 (45.35%)	6707603 (47.86%)
Reads mapped in proper pairs	14680818 (86.66%)	11723158 (87.61%)	10259114 (80.73%)	14083462 (88.17%)	13387146 (85.46%)	12026152 (85.97%)	14291416 (88.22%)	14902196 (82.29%)	12166502 (86.82%)
Proper-paired reads map to different chrom	0 (0%)	0 (0%)	0 (0%)	0 (0%)	0 (0%)	0 (0%)	0 (0%)	0 (0%)	0 (0%)

Supporting Information Table S5: RT-qPCR primers used in this study.

Gene	Forward	Reverse	Efficiency	Reference
16S	CTGTGCACATCTTGACGGTA	TCAGCGTCAGTTACAGACCA	93.80%	Yarwood et al.[1]
hla	ATGAATCCTGTCGCTAATGCCG	TGACCAGCAATGGTACCTTTCG	107.02%	This work
hlgA	AGCAGTTGGTTTAATCGCCCCTTT	TTGATGATTTCTGCACCTTGGCCG	83.86%	Viering et al.[2]
hlgC	GGTGGTAATTTCCAATCAGCC	GAATGAATTCGCTTTGACGCCC	109.18%	This work

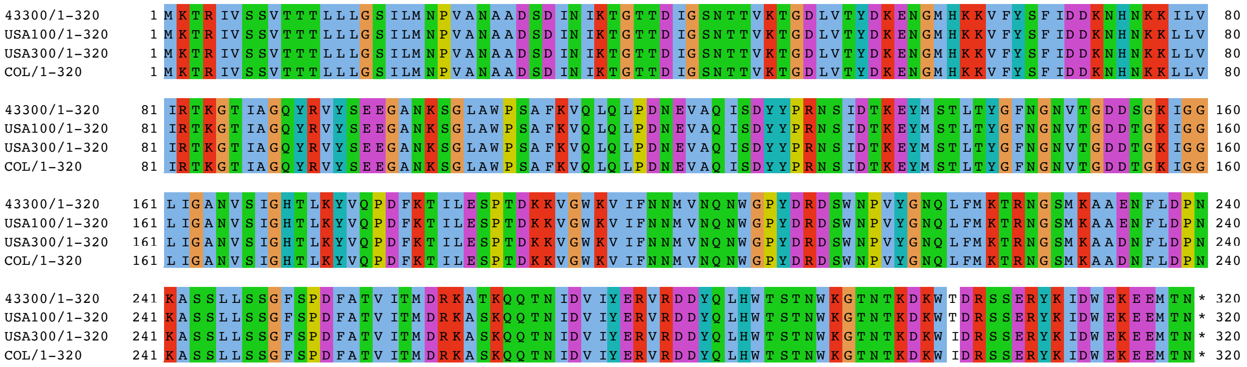

Supporting Figure S1: Alpha hemolysin precursor is highly conserved among analyzed MRSA strains. Amino acids are colored based on Clustal settings.

A)

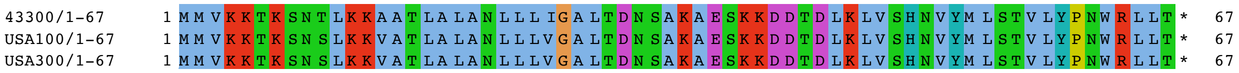

B)

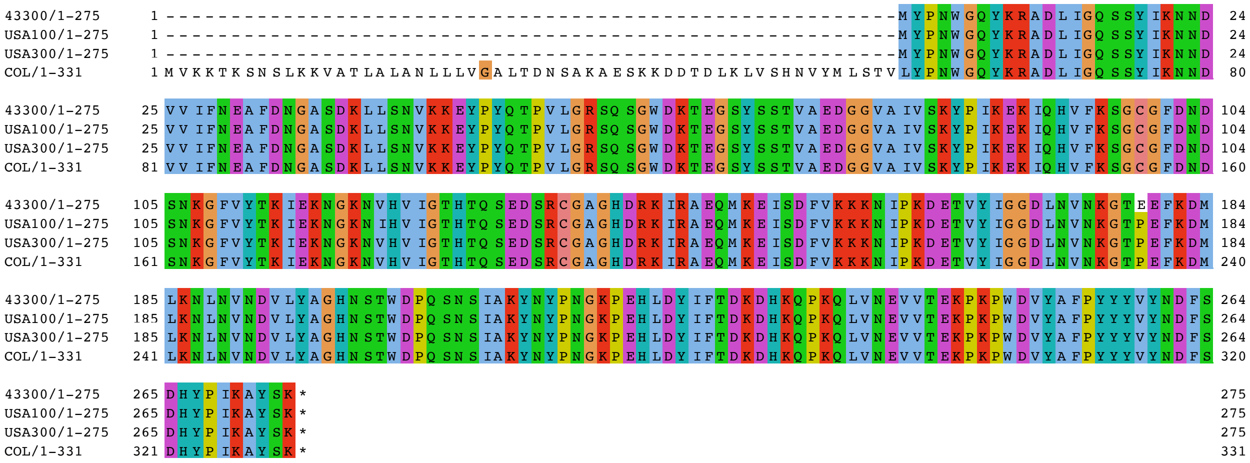

Supporting Figure S2: Beta hemolysin is highly conserved among analyzed MRSA strains. In both panels, amino acids are colored based on Clustal settings. A) The sequences are identical in the upstream instance (amino acids 1-67). B) This gene is disrupted by a prophage in MRSA strains 43300, USA100, and USA300, but the highly conserved downstream instance is shown.

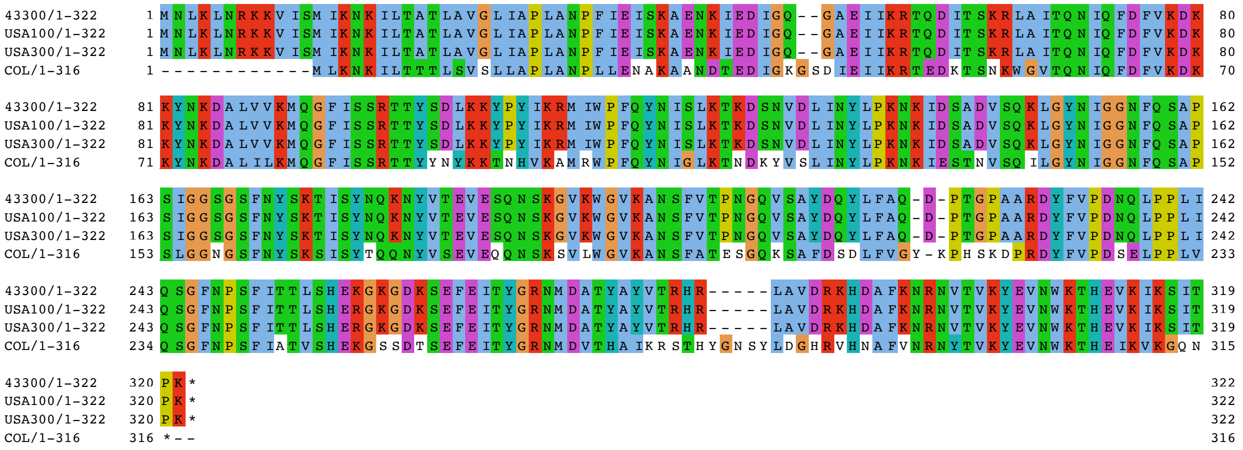

Supporting Figure S3: Gamma hemolysin component A is highly conserved among 43300, USA100 and USA100. Amino acids are colored based on Clustal settings.

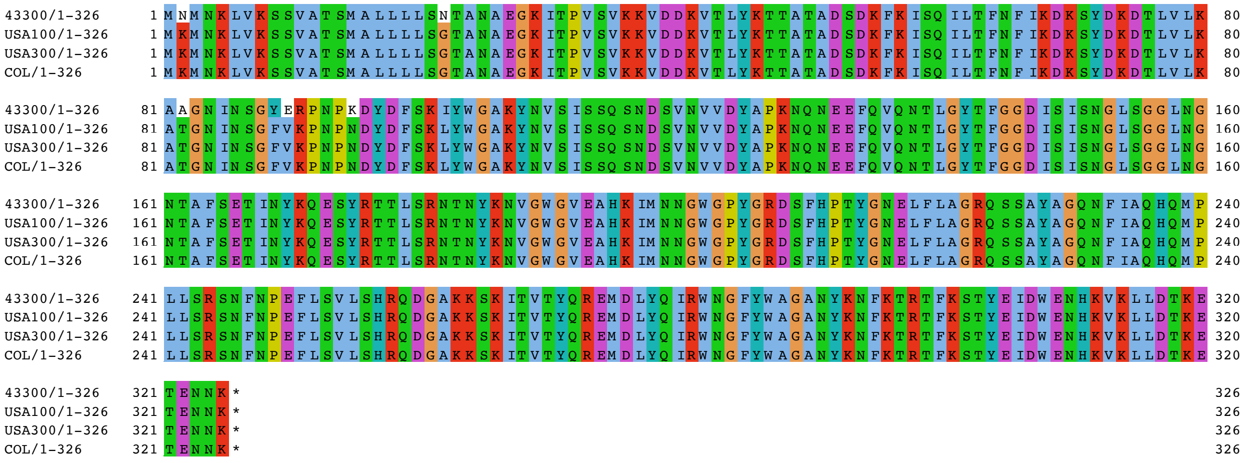

Supporting Figure S4: Gamma hemolysin component B is highly conserved among analyzed MRSA strains. Amino acids are colored based on Clustal settings.

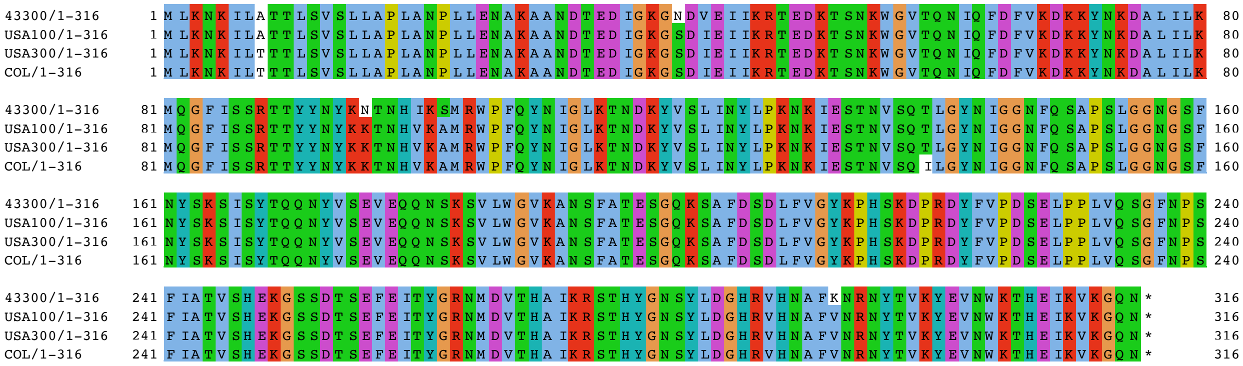

Supporting Figure S5: Gamma hemolysin component C is highly conserved among analyzed MRSA strains. Amino acids are colored based on Clustal settings.

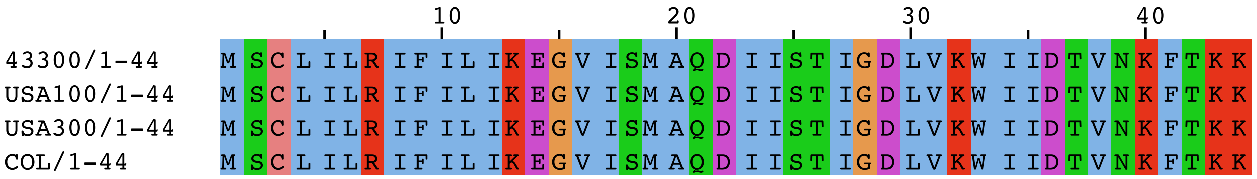

Supporting Figure S6: Delta hemolysin precursor is identical in analyzed MRSA strains. Amino acids are colored based on Clustal settings.

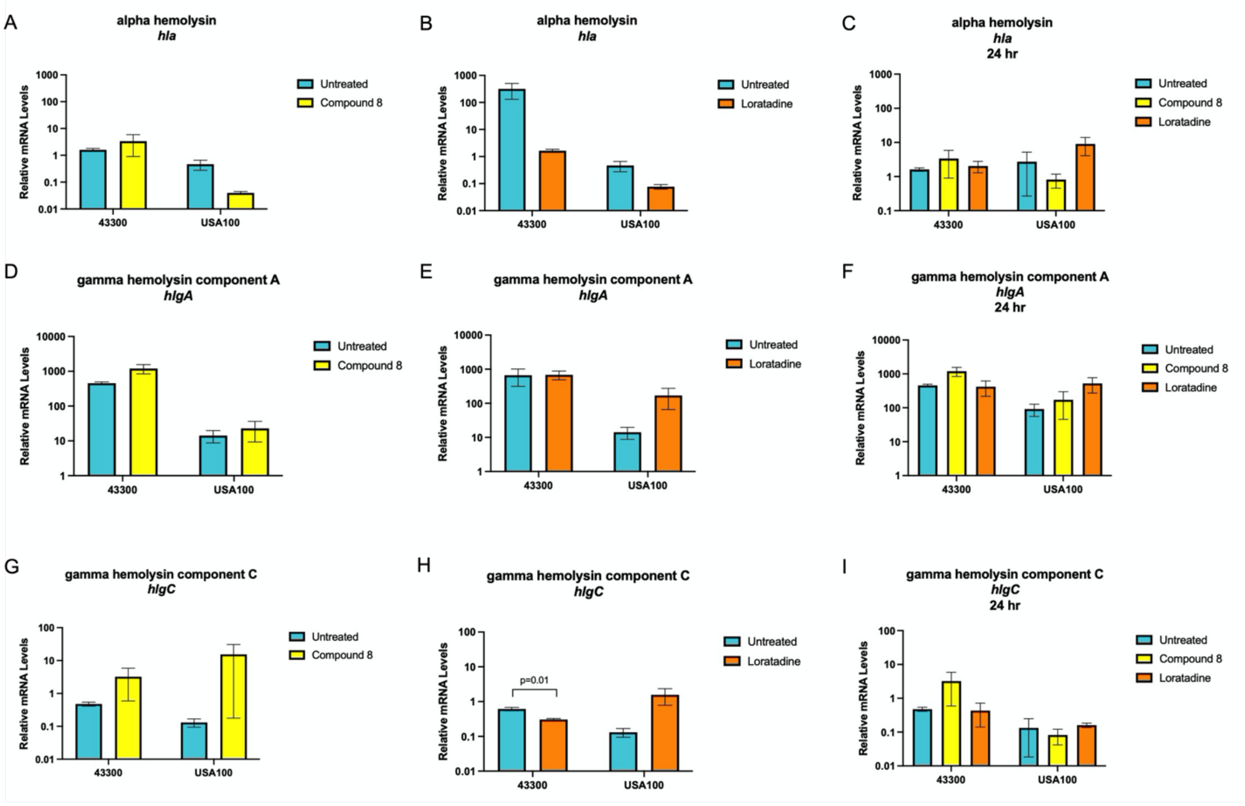

Supporting Figure S7: RT-qPCR validation of RNA-seq determined gene expression changes. Results are the mean of at least three biological replicates. The y axis displays the average mRNA levels relative to *16S* rRNA. Error bars represent the standard error of the mean. Statistical significance was analyzed by students unpaired t-tests between treated and untreated levels. p values ≤ 0.05 are indicated.

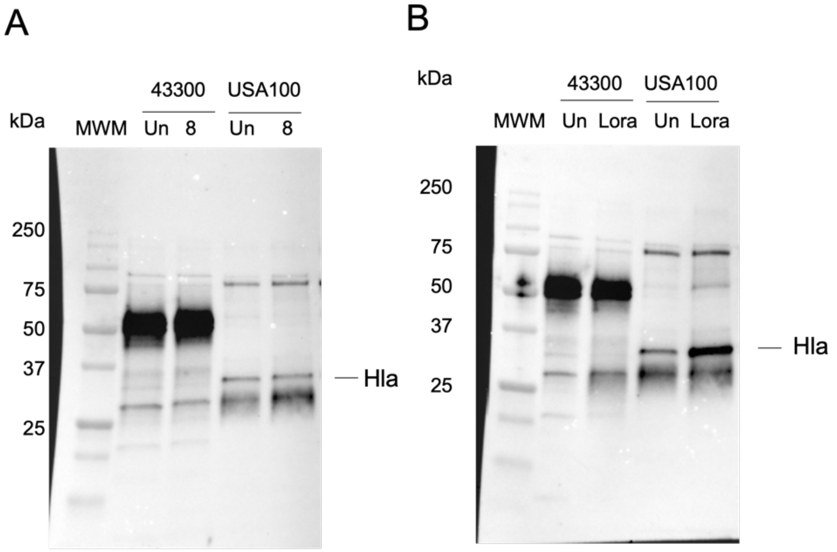

Supporting Figure S8: Levels of secreted alpha hemolysin are affected by compound 8 and loratadine treatment. A) Full western blot image of that shown in Figure 4A. B) Full western blot image of that shown in Figure 4B. In all panels, MWM is molecular weight marker, Un is untreated, 8 is compound 8, and Lora is loratadine.

References

(1) Yarwood, J. M.; McCormick, J. K.; Paustian, M. L.; Kapur, V.; Schlievert, P. M. Repression of the Staphylococcus aureus accessory gene regulator in serum and in vivo. *J Bacteriol* **2002**, *184* (4), 1095-1101. DOI: 10.1128/jb.184.4.1095-1101.2002.
(2) Viering, B.; Cunningham, T.; King, A.; Blackledge, M. S.; Miller, H. B. Brominated carbazole with antibiotic adjuvant activity displays pleiotropic effects in MRSA's transcriptome. *ACS Chem Biol* **2022**, *17* (5), 1239-1248. DOI: 10.1021/acschembio.2c00168 From NLM Medline.
